# Teaching clinical practical and communication skills of the clinical skills lab of the University of Veterinary Medicine Hannover, Foundation, Germany during the COVID-19 pandemic

**DOI:** 10.3205/zma001482

**Published:** 2021-06-15

**Authors:** Silja Brombacher-Steiert, Raphaela Ehrich, Claudia Schneider, Lina R. Müller, Andrea Tipold, Sandra Wissing

**Affiliations:** 1 University of Veterinary Medicine Hannover, Foundation, Centre for E-Learning, Didactics and Training Research, Clinical Skills Lab, Hannover, Germany; 2 University of Veterinary Medicine Hannover, Foundation, Centre for E-Learning, Didactics and Training Research, E-Learning Consulting, Hannover, Germany; 3 University of Veterinary Medicine Hannover, Foundation, Clinic for Small Animals, Hannover, Germany

**Keywords:** veterinary medicine, digital teaching, moodle, online teaching format, practical skills, blended learning.

## Abstract

**Objective: **The aim of the project is to teach clinical practical and communication skills in the Clinical Skills Lab (CSL) of the University of Veterinary Medicine Hannover, Foundation (TiHo) during the COVID-19 pandemic.

**Methodology: **As a measure to limit potential SARS-CoV2 infections, the CSL learning stations were digitised and made available on the Moodle learning platform of the TiHo. Online quiz stations were also developed, as well as improvisations that allowed students to practise practical skills at home using everyday materials. Courses for Practical Year (PY) students were digitised and again combined with classroom exercises throughout the year. The teaching formats could be evaluated by the students by means of a questionnaire using a Likert scale (1=agree; 4=disagree).

**Results:** A total of 24.92% of students (n=1272) completed the learning stations with improvisations. The quiz stations were completed with a percentage of 75.08%. Students indicated that the improvisations were easily implementable from home (M=1.33) and assisted in learning the practical skills in question (M=1.89). The quiz stations were considered helpful (M=1.40) and complementary to previous CSL offers (M=1.13). The PY students found the amount of teaching materials adequate (M=1.76) and described communication with the lecturers as problem-free (compulsory electives=1.24).

**Conclusions: **Digital teaching is suitable as a supplement to existing face-to-face courses at the CSL, but cannot replace on-site training under the guidance of trained personnel. The CSL will continue to strive for a combination of online and face-to-face courses for some learning stations in the future.

## Zusammenfassung

**Zielsetzung: **Ziel des Projekts ist die Vermittlung klinisch-praktischer und kommunikativer Fertigkeiten im Clinical Skills Lab (CSL) der Stiftung Tierärztliche Hochschule Hannover (TiHo) während der COVID-19-Pandemie.

**Methodik: **Als Maßnahme zur Einschränkung möglicher Infektionen durch SARS-CoV2 wurden die Lernstationen des CSL digitalisiert und auf der Lernplattform Moodle bereit gestellt. Außerdem wurden Online-Quizstationen sowie Improvisationen entwickelt, bei denen Studierende praktische Fertigkeiten zu Hause mit alltäglichen Materialien trainieren konnten. Kurse für Studierende des Praktischen Jahres (PJ) wurden digitalisiert und im Verlauf des Jahres wieder mit Präsenz-Übungen kombiniert. Die Lehrformate konnten von den Studierenden mittels Fragebogen anhand einer Likert-Skala (1= trifft zu bis 4= trifft nicht zu) evaluiert werden.

**Ergebnisse: **24,92% der Studierenden (n=1272) führten die Lernstationen mit Improvisationen durch. Die Quizstationen wurden mit einem Anteil von 75,08 % absolviert. Die Studierenden gaben an, dass die Improvisationen problemlos von zu Hause aus umsetzbar waren (MW=1,33) und dabei unterstützten, die betreffenden praktischen Fertigkeiten zu erlernen (MW: 1,89). Die Quizstationen wurden als hilfreich (MW=1,40) und als Ergänzung zu den bisherigen Angeboten des CSL (MW=1,13) angesehen. Die PJ-Studierenden fanden den Umfang der Lehrmaterialien angemessen (MW=1,76) und beschrieben die Kommunikation mit den Dozierenden als problemlos (WP=1,24).

**Schlussfolgerungen:** Digitale Lehre eignet sich als Ergänzung zu bestehenden Präsenzkursen des CSL, kann das Training vor Ort unter Anleitung von geschultem Personal aber nicht ersetzen. Eine Kombination aus Online-und Präsenzveranstaltungen wird vom CSL auch in Zukunft für einige Lernstationen angestrebt.

## 1. Introduction

Since 2013, the Clinical Skills Lab (CSL) of the University of Veterinary Medicine Hannover Foundation (TiHo) has been teaching practical and communication skills to veterinary students. Annually, an average of 1,780 students attend the institute to practice their skills in optional learning stations [1]. Until March 2020, skills training took place in peer-group teaching with an emphasis on hands-on learning exclusively as face-to-face sessions. 

Due to the COVID-19 pandemic and associated constraints [2], the CSL developed ways to teach competencies that do not require face-to-face contact but are digitally applicable via the online learning platform Moodle used at the TiHo. Moodle offers a variety of design options for digital teaching, such as providing teaching and information material, testing performance, and the possibility of giving feedback and conducting evaluations, and was thus well suited for the implementation of this project. Another reason was to be able to give as many students as possible the opportunity to participate in the digital teaching formats.

Other national veterinary institutions also established digital skills labs [3] and good communication exchange took place [4].

## 2. Project description

Of a total of 44 CSL stations, 42 were digitally prepared for online courses on Moodle. Students were able to flexibly access all instructions, worksheets with questions for self-examination, instructional videos, and further information material from the CSL and work on them independently. 

Sixteen CSL Moodle stations were supplemented by so-called “improvisations”, demonstrating to students how they could implement the exercises at home using everyday materials according to the “do-it-yourself” principle. Eleven cross-topic quiz stations complemented the online offer. 

The courses for students in the practical year (PY) were initially implemented online and in the course of the year as hybrid events.

### 2.1. Digital learning stations with improvisations

**Figure 1:**
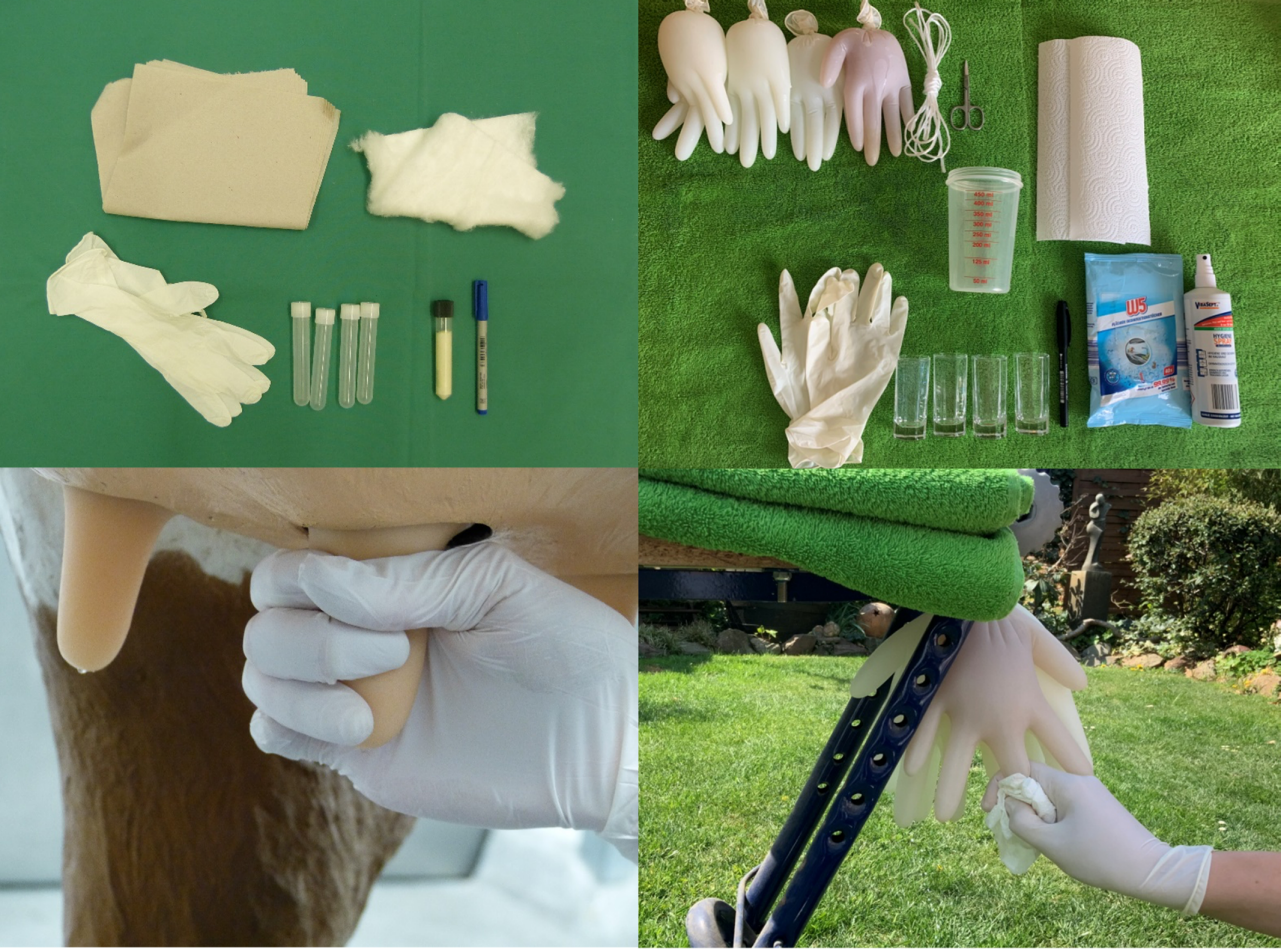
Comparison of the original (top left and bottom of picture) and the digital (top right and bottom of picture) CSL learning station "milk sampling in cattle.”

The improvisations of the moodle stations contain detailed information on how students can learn or consolidate practical skills at home using everyday objects in self-study. To do this, students must make the described model themselves, perform the exercise independently, document it photographically and upload it to Moodle, and work on the associated worksheet (see figure 1 (Fig. 1)). The results are evaluated by teaching staff and the students receive feedback.

#### 2.2. Cross-thematic quiz stations

In addition to learning stations with improvisations, cross-topic quiz stations were developed. An average of 20 multiple-choice questions were generated per quiz station. The instructions provided serve as a basis for answering the quizzes and must be worked through in advance. A quiz is considered passed if at least 60% of the questions are answered correctly. 

#### 2.3. Training for students in their practical year

**Figure 2:**
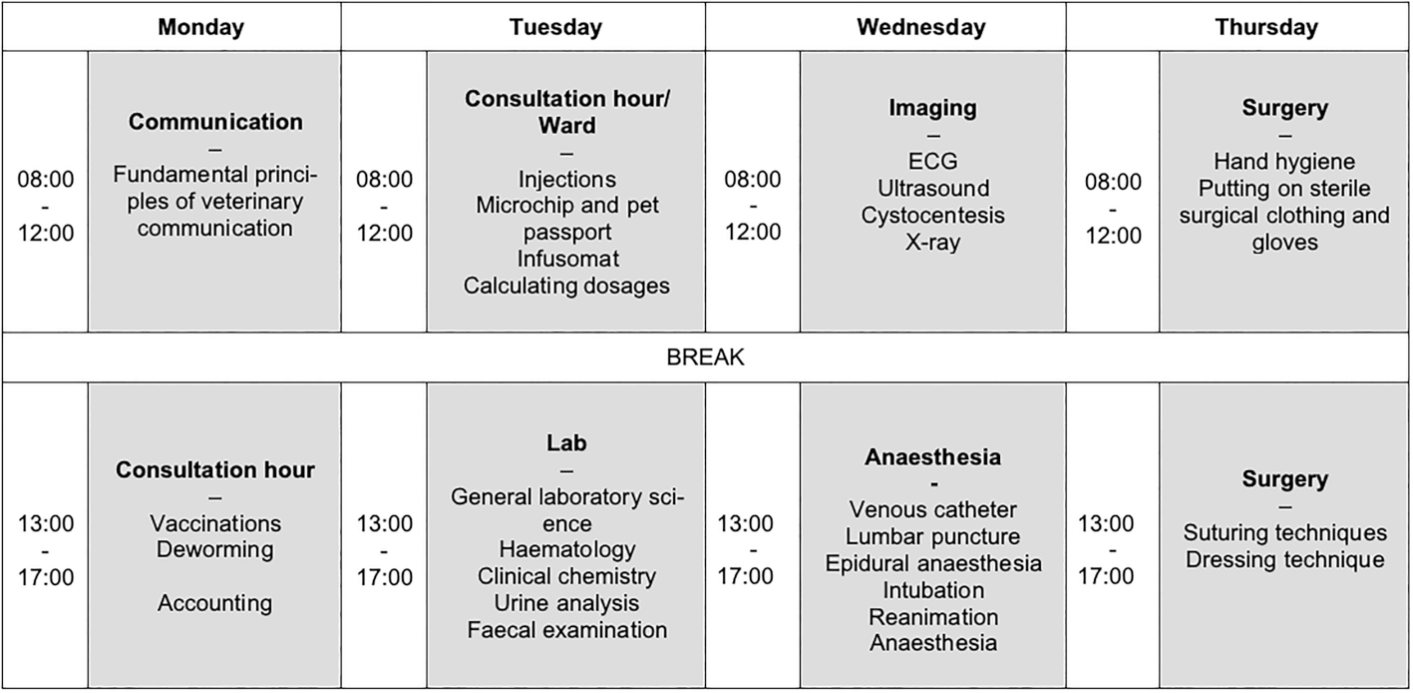
Weekly plan for the digital training in the CSL for practical year students of the Clinic for Small Animals. The commnication topic block took place via Microsoft Teams, the remaining content via Moodle.

In April 2020, the training week for PY students at the CSL took place exclusively online for the first time (see figure 2 (Fig. 2)). A total of 21 students participated in preparation for their PY at the Small Animal Clinic. Learning materials relevant to practice were provided asynchronously on Moodle and learning successes were checked via worksheets and free text submissions. The lecture on Veterinary Communication was delivered synchronously via MS teams and provided opportunities for interactive discussion. The following trainings took place digitally with the inclusion of face-to-face sessions. 

#### 2.4. Evaluating the project

The online learning formats were activated for students in May 2020. The number of completed learning stations, for which the worksheet as well as the improvisation were submitted, and the number of successfully completed quiz stations in the period from the beginning of May to mid-October 2020 were evaluated.

Since the end of September 2020, students have been able to provide anonymous feedback on the digital learning and quiz stations as well as the courses of the practical year using an online questionnaire with the Likert scale and free text information. 

## 3. Results

In the above period, 317 students participated in the learning stations and 955 students in the quiz stations. In comparison, 807 students completed the corresponding learning stations in face-to-face teaching in 2019 during the same period (see table 1 (Tab. 1)).

To date, a total of 24 feedback forms could be evaluated, nine of them for the learning stations and 15 for the quiz stations (see table 2 (Tab. 2) and table 3 (Tab. 3)). 

## 4. Discussion

The presented teaching formats offer students the opportunity to acquire knowledge analogous to the levels of Miller’s pyramid [5] (“knows”) and to put it into practice (“knows how”) and demonstrate it (“shows”) in the context of the improvisations. In order to keep the number of participants large and to offer time flexibility, asynchronous teaching formats were deliberately used. Peer group teaching via MS teams was ruled out due to staff resources and feasibility for large groups, but was successfully used to train new CSL staff in specific stations (e.g. suturing techniques) using the Peyton Approach. 

Digital teaching enables skills transfer during the pandemic while contributing to infection control by avoiding direct interpersonal contact. However, not all CSL learning stations are suitable for use in digital teaching. Simple hands-on skills are easy to perform with common household materials (e.g., knotting techniques). However, practising complex skills (e.g. intubation) requires specialised models that are not easily replicated. Necessary materials may also be difficult to obtain (e.g. venous catheters). This is also reflected in the lower participation rates of the online stations compared to the face-to-face learning stations. In addition, there is no immediate error correction by teaching staff. Due to these disadvantages, it is questionable whether students can achieve similar learning successes by means of online learning stations as with exercises on commercial simulators or live animals.

## 5. Conclusions

The teaching of competencies through online teaching formats is a useful addition to the existing CSL offer. Students can prepare well for classroom events with digital learning materials. However, digital teaching cannot replace the teaching of practical skills on site by trained personnel. 

Therefore, a combination of face-to-face and online teaching (blended learning) was introduced at CSL in July. Since then, face-to-face courses have been held in small groups under hygienic conditions. In addition, online teaching formats on Moodle remain in place. 

In order to further optimise digital teaching, the evaluation of the online teaching formats will be continued. Further collaboration with skills labs of other veterinary institutions is sought, to expand the online learning stations.

## Competing interests

The authors declare that they have no competing interests. 

## Figures and Tables

**Table 1:**
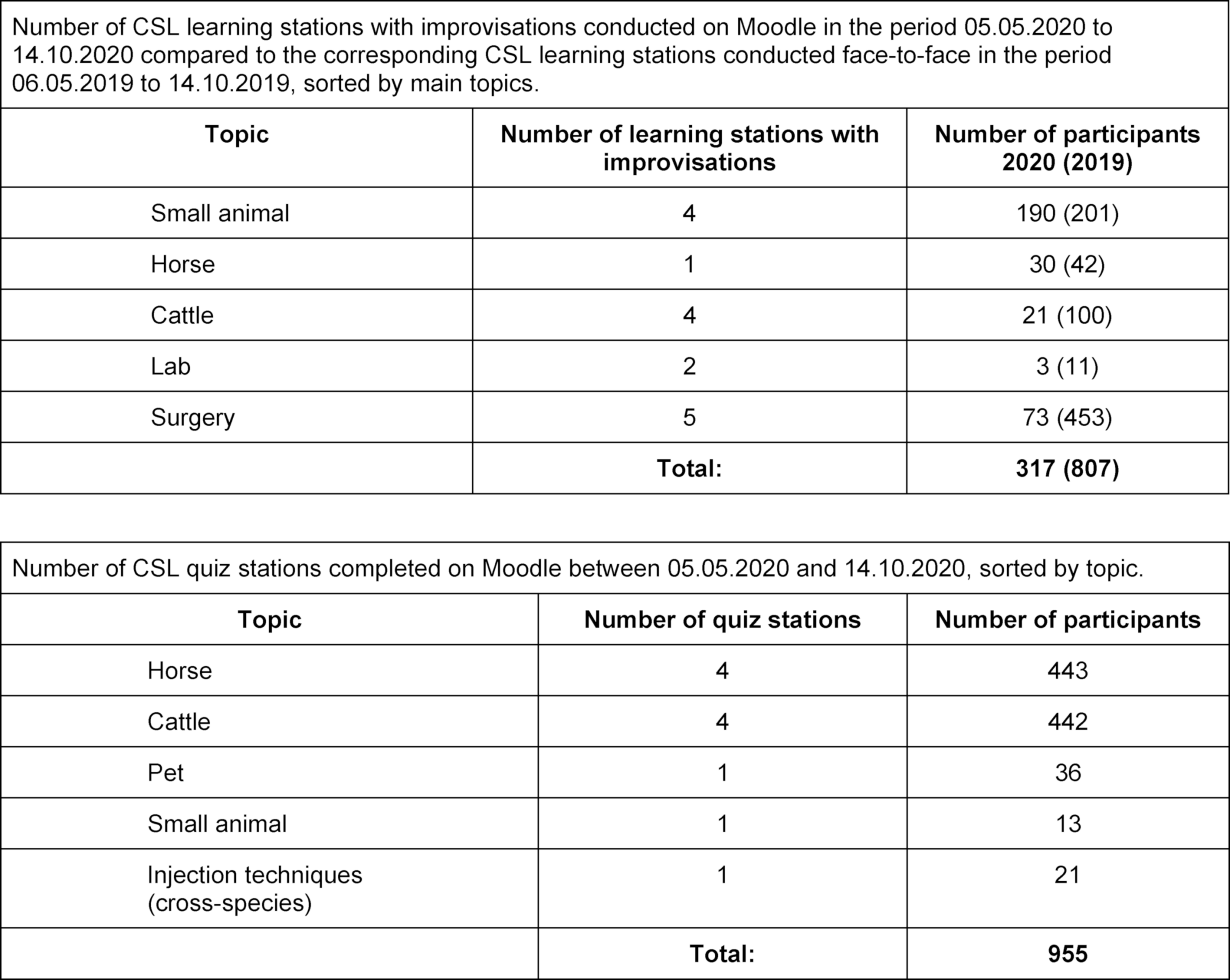
Number of CSL teaching stations and CSL quiz stations completed on Moodle between 05.05.2020 and 14.10.2020.

**Table 2:**
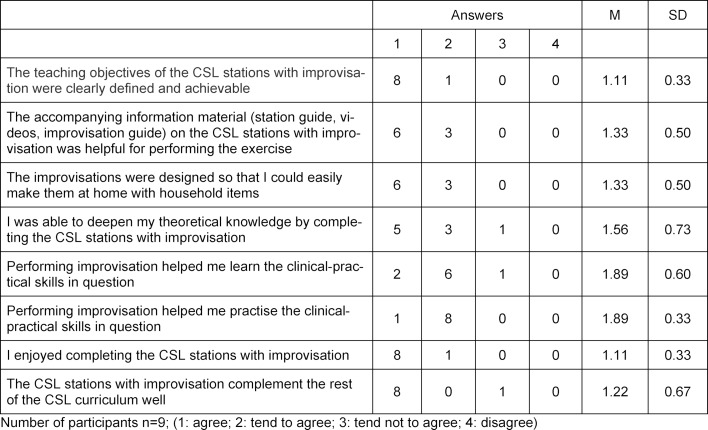
Excerpt of student feedback on CSL learning stations with improvisation.

**Table 3:**
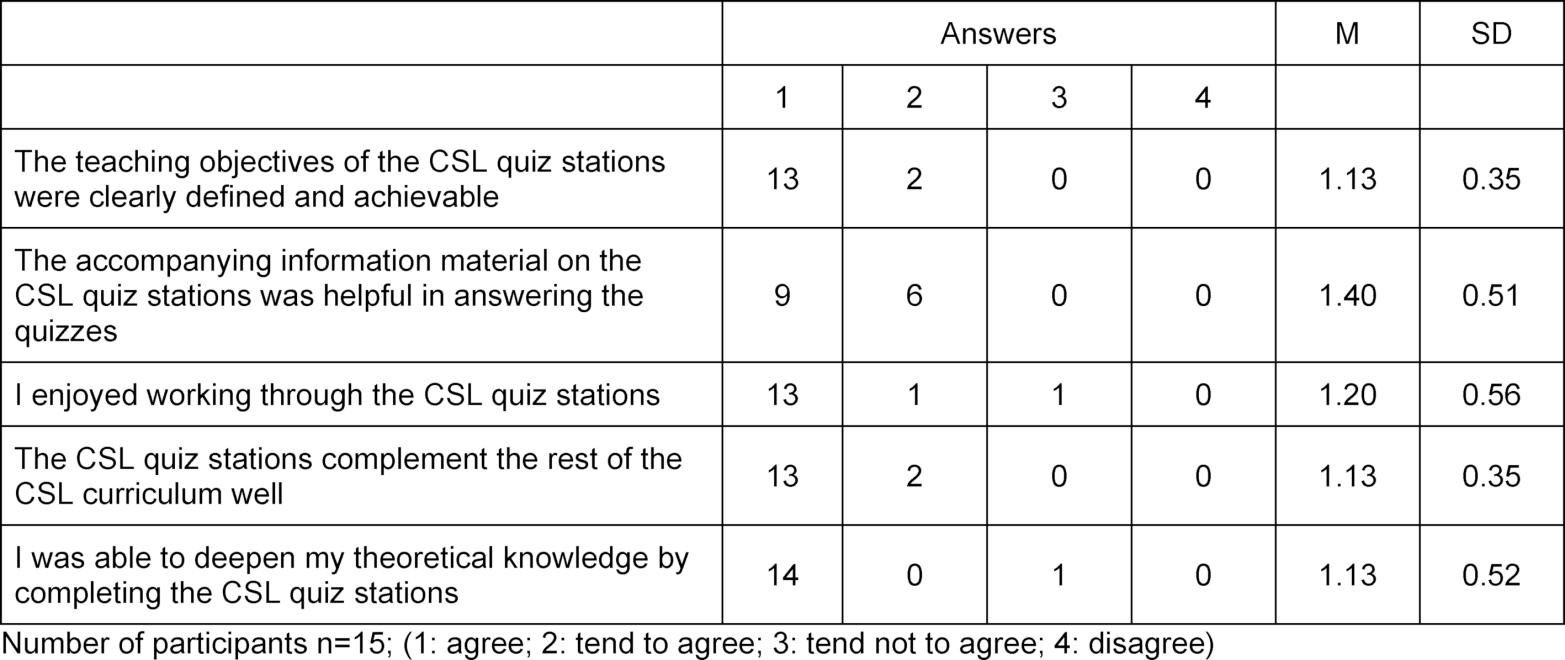
Excerpt from student feedback on CSL quiz stations.
